# Clinical gradient and predictors for stepwise resistance escalation of carbapenem-susceptible, carbapenem-resistant, and difficult-to-treat resistant *Pseudomonas aeruginosa* infections: a 6-year retrospective cohort study

**DOI:** 10.3389/fcimb.2026.1930952

**Published:** 2026-09-14

**Authors:** Lin Chen, Donghong Yin, Huimin Hu, Jianbang Kang, Renaguli Rouzimaimaiti, Jinju Duan, Ruigang Hou

**Affiliations:** 1 School of Pharmacy, Shanxi Medical University, Taiyuan, Shanxi, China; 2 Medicinal Basic Research Innovation Center of Chronic Kidney Disease, Ministry of Education, Shanxi Medical University, Taiyuan, Shanxi, China; 3 Department of Pharmacy, Second Hospital of Shanxi Medical University, Taiyuan, Shanxi, China

**Keywords:** antimicrobial resistance, difficult-to-treat resistant Pseudomonas aeruginosa (DTR-PA), resistance acquisition, resistance escalation, risk factors

## Abstract

**Objective:**

Difficult-to-treat resistant *Pseudomonas aeruginosa* (DTR-PA) represents the most severe antimicrobial resistance phenotype of *P. aeruginosa*, with limited therapeutic options and poor clinical outcomes. However, previous studies have largely focused on carbapenem-resistant *P. aeruginosa* (CRPA), and the factors associated with resistance acquisition and progression remain insufficiently characterized. This study aimed to investigate the clinical characteristics and risk factors associated with different resistance phenotypes of *P. aeruginosa* from the perspectives of both resistance acquisition and resistance escalation.

**Methods:**

A retrospective case-control study was conducted from January 2020 to December 2025 at a tertiary teaching hospital. *P. aeruginosa* isolates were classified according to antimicrobial susceptibility profiles. All DTR-PA cases (n = 67) were included, and CRPA and carbapenem-susceptible *P. aeruginosa* (CSPA) controls were selected using a time-stratified 1:2:2 sampling strategy. Clinical data were collected, and three comparative models were developed to explore determinants of resistance acquisition and resistance escalation across different *P. aeruginosa* phenotypes: resistant *P. aeruginosa* (R-PA, defined as CRPA and DTR-PA) versus CSPA, DTR-PA versus CRPA, and DTR-PA versus non-DTR-PA.

**Results:**

During the study period, 2,692 *P. aeruginosa* clinical isolates were included for analysis, including 67 (2.5%) DTR-PA, 407 (15.1%) CRPA, and 2,218 (82.4%) CSPA. A total of 335 patients were included in the risk factor analysis. With increasing antimicrobial resistance severity, inflammatory markers showed a stepwise increase, whereas nutritional status, as reflected by serum albumin levels, demonstrated a progressive decline. In the R-PA versus CSPA model, invasive respiratory procedures, prolonged hospitalization, and polymicrobial co-infection were associated with increased risk, whereas higher BMI and serum albumin levels showed modest inverse associations with resistant *P. aeruginosa* infection. In the DTR-PA versus CRPA model, diabetes mellitus, chronic respiratory diseases, and recent ICU admission were associated with DTR-PA infection. In the DTR-PA versus non-DTR-PA model, prior antibiotic exposure, central venous catheterization, and invasive procedures were associated with DTR-PA infection.

**Conclusion:**

Antimicrobial resistance in *P. aeruginosa* follows a dynamic, stage-dependent process with distinct risk profiles. Healthcare exposure primarily contributes to resistance emergence, whereas host comorbidities and intensive care exposure are more closely associated with progression to DTR-PA. This resistance gradient framework may support early identification, risk stratification, and infection control strategies.

## 1 Introduction

*Pseudomonas aeruginosa* is a clinically important opportunistic pathogen and one of the most common Gram-negative bacteria responsible for healthcare-associated infections, including pneumonia, bloodstream infections, intra-abdominal infections, and urinary tract infections. According to the China Antimicrobial Surveillance Network (CHINET) 2025 report, *P. aeruginosa* ranks fourth among clinical bacterial isolates in China (Chinet, 2025). Antimicrobial resistance (AMR) has emerged as a major global public health threat, responsible for an estimated 1.14 million deaths directly and 4.71 million associated deaths worldwide in 2021. Without effective interventions, AMR may cause 1.91 million direct and 8.22 million associated deaths annually by 2050. P*. aeruginosa* has been identified as one of the key Gram-negative pathogens contributing substantially to AMR-related mortality (Antimicrobial Resistance Collaborators, 2022; GBD 2021 Antimicrobial Resistance Collaborators, 2024).

Owing to its environmental adaptability and a combination of intrinsic and acquired resistance mechanisms, carbapenem-resistant *P. aeruginosa* (CRPA) has become a major clinical concern. In the 2024 updated WHO priority pathogen list, CRPA was classified as a high-priority pathogen, underscoring its global public health threat (Balakrishnan, 2022).

To better characterize limitations in clinical treatment options, antimicrobial resistance phenotypes have been increasingly standardized. Traditional definitions of multidrug-resistant (MDR), extensively drug-resistant (XDR), and pandrug-resistant (PDR) bacteria are based on *in vitro* antimicrobial susceptibility profiles. However, these classifications do not directly reflect the extent of clinical treatment limitations (Magiorakos et al., 2012).

In 2018, Kadri et al. proposed the concept of difficult-to-treat resistance (DTR), defining DTR *P. aeruginosa* (DTR-PA) as non-susceptibility to all of the following key first-line antipseudomonal agents: piperacillin-tazobactam, ceftazidime, cefepime, aztreonam, meropenem, imipenem-cilastatin, ciprofloxacin, and levofloxacin (Kadri et al., 2018). Compared with traditional MDR/XDR definitions, DTR more accurately reflects the clinical reality of extremely limited or absent effective therapeutic options.

In recent years, DTR-PA has received increasing attention as an emerging resistant phenotype. Reported detection rates vary widely (3.5%–15.3%) of all clinical *P. aeruginosa* isolates (Itani et al., 2025a; Shabi et al., 2025). Global surveillance data indicate that DTR phenotypes have persisted among key Gram-negative pathogens prioritized by the World Health Organization between 2018 and 2022, with an increasing trend observed in multiple regions (Wise et al., 2024). More importantly, due to its non-susceptibility to key first-line antipseudomonal agents, DTR-PA infections are frequently associated with delays in the initiation of effective empirical antimicrobial therapy, thereby leading to worse clinical outcomes. A multicenter study in four Lebanese tertiary teaching hospitals showed that DTR-PA infection was independently associated with increased risk of 30-day mortality (AOR 2.48, 95% CI 1.32-4.63) (Itani et al., 2025a). Given its extremely limited treatment options and high mortality risk, DTR-PA has become a major challenge in current clinical infection management.

At present, most studies on antimicrobial resistance in *P. aeruginosa* have focused on the epidemiological characteristics and risk factors of CRPA, while evidence specifically addressing DTR-PA remains relatively limited. Existing studies have largely adopted a two-group comparison design between DTR-PA and non-DTR-PA (Itani et al., 2025a; Itani et al., 2025b), lacking systematic analyses across the full resistance spectrum from carbapenem-susceptible *P. aeruginosa* (CSPA) to CRPA, and further to DTR-PA. Therefore, based on *P. aeruginosa* clinical isolates from a large tertiary teaching hospital, this study aimed to determine the detection rate and resistance evolution of DTR-PA by establishing a three-tier cohort (CSPA, CRPA, and DTR-PA) and analyzing clinical characteristics and risk factors across resistance emergence and escalation. The findings provide evidence for early identification of high-risk DTR-PA patients and support infection control and antimicrobial stewardship strategies.

## 2 Methods

### 2.1 Study design

We conducted a retrospective cohort study using a bacterial isolation and antimicrobial resistance database established by the Clinical Microbiology Laboratory of the Second Hospital of Shanxi Medical University. Patients with microbiologically confirmed *P. aeruginosa* infection between January 1, 2020, and December 31, 2025, were screened for inclusion. The exclusion criteria were: (1) outpatient specimens; (2) missing key antimicrobial susceptibility data. For microbiological de-duplication, repeated antimicrobial susceptibility reports of *P. aeruginosa* obtained from the same patient and the same specimen source no more than 3 days apart were considered to represent the same microbiological episode, and only the first report was retained. The study was approved by the institutional ethics committee. The requirement for informed consent was waived due to the retrospective design and anonymization of data. The study followed STROBE guidelines (Von Elm et al., 2007).

Isolates were categorized into three phenotypes according to antimicrobial susceptibility profiles: DTR-PA, CRPA, and CSPA. To ensure that each patient contributed only one observation, all eligible antimicrobial susceptibility records were manually reviewed and de-duplicated at the patient level according to a predefined phenotype hierarchy: DTR-PA, followed by CRPA and then CSPA. For each patient, the highest resistance phenotype documented during the study period was retained. Accordingly, patients with at least one DTR-PA isolate were classified as DTR-PA; patients without DTR-PA but with at least one CRPA isolate were classified as CRPA; and patients with only CSPA isolates were classified as CSPA.

If the selected phenotype was identified during multiple hospitalizations, the most recent hospitalization in which that phenotype occurred was retained. Within the retained hospitalization, the first antimicrobial susceptibility report meeting the definition of the selected phenotype was used to identify the representative isolate for the risk-factor analysis. Each retained record therefore represented one unique patient.

After deduplication, prevalence and temporal trends of DTR-PA were assessed, and antimicrobial susceptibility patterns were summarized by phenotype. For risk factor analysis, all patients classified as DTR-PA were included. CRPA and CSPA were selected using stratified random sampling by year and quarter at a 1:2:2 ratio.

### 2.2 Definitions

DTR-PA was defined according to the Infectious Diseases Society of America (IDSA) criteria as non-susceptibility to all of the following agents: aztreonam, piperacillin–tazobactam, ceftazidime, cefepime, imipenem, meropenem, ciprofloxacin, and levofloxacin (Tamma et al., 2023). CRPA was defined according to the Clinical and Laboratory Standards Institute (CLSI) M100 interpretive criteria as resistance to at least one carbapenem (imipenem or meropenem), whereas CSPA was defined as susceptibility to both agents (CLSI, 2025). Resistant *P. aeruginosa* (R-PA) was defined as the combined group of CRPA and DTR-PA. Critical illness was defined as an explicit designation of critical illness in the discharge record for the retained hospitalization. ICU admission during the current hospitalization was defined as any admission or transfer to an intensive care unit during that hospitalization. Times of hospitalizations referred to how many times the patient had been admitted to our hospital, including the retained hospitalization.

### 2.3 Data collection

Clinical data were extracted from the hospital electronic medical record system. The collected variables were categorized into six domains: (1) demographic and clinical characteristics, including sex, age, body mass index (BMI), department, and number of hospital admissions; (2) host factors and prior medication exposure, including special medication exposure (corticosteroids, immunosuppressants, SGLT2 inhibitors, and advanced antibiotics) and underlying medical conditions; (3) healthcare exposure and disease severity, including disease severity, ICU admission during the current hospitalization, healthcare exposure within the previous 3 months (hospitalization, ICU stay, and antibiotic exposure), healthcare utilization (length of hospital stay and hospitalization costs), and invasive procedures; (4) laboratory parameters, including liver function, renal function, and inflammatory markers; (5) infection-related characteristics, including infection sites and non-bacterial co-infections; and (6) microbiological characteristics, including bacterial co-isolates and specimen types.

### 2.4 Statistical analysis

All data were organized using Microsoft Excel 2019 and analyzed using R (version 4.3.3). Continuous variables were presented as mean [standard deviation (SD)] or median [interquartile range (IQR)], as appropriate. Continuous variables presented as mean (SD) were compared among the three groups using one-way analysis of variance, whereas those presented as median (IQR) were compared using the Kruskal–Wallis test. Categorical variables were presented as number (percentage) and compared using Pearson’s chi-square test. Variables with more than 20% missing data were excluded, and the remaining missing values were handled using multiple imputation (m = 20). Univariate logistic regression and LASSO regression were performed for risk factor analysis and variable selection. Multivariable analysis was conducted using Firth’s penalized logistic regression. Model discrimination and calibration were evaluated using the area under the receiver operating characteristic curve (AUC) and the Hosmer-Lemeshow test. Sensitivity analyses were performed using complete-case and multiple-imputation datasets. A two-sided p < 0.05 was considered statistically significant.

## 3 Results

### 3.1 Isolation and antimicrobial resistance profiles of *P. aeruginosa* in our hospital

During the period from January 1, 2020, to December 31, 2025, a total of 2,692 *P. aeruginosa* isolates recovered from clinical specimens of hospitalized patients were identified in our hospital. Of these, 67 (2.5%) were classified as DTR-PA, 407 (15.1%) as CRPA, and 2,218 (82.4%) as CSPA (**Figure 1**).

**Figure 1:**
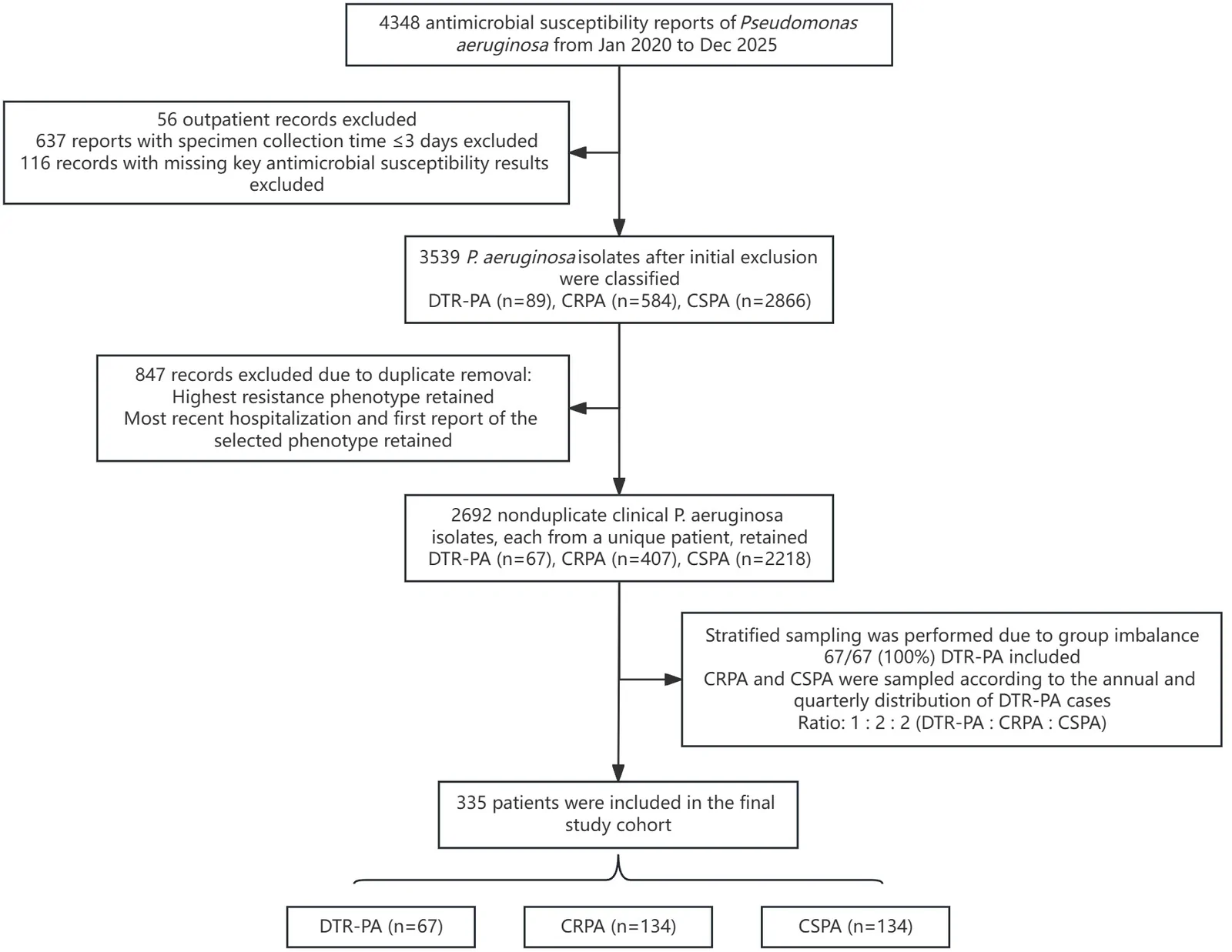
Flow diagram of *P. aeruginosa* isolate selection, classification, and study cohort construction. CSPA, carbapenem-susceptible *Pseudomonas aeruginosa;* CRPA, carbapenem-resistant *P. aeruginosa*; DTR-PA, difficult-to-treat resistant *P. aeruginosa*.

Clinical isolates were predominantly obtained from respiratory tract specimens, with sputum and bronchoalveolar lavage fluid accounting for the highest proportion, followed by urine specimens, while blood-derived isolates represented a relatively small proportion. Overall, CSPA remained the predominant phenotype throughout the study period. Notably, the proportion of DTR-PA increased between 2020 and 2022 and subsequently stabilized, suggesting a possible plateau in the nosocomial spread of highly resistant strains in our hospital (**Figure 2**).

**Figure 2:**
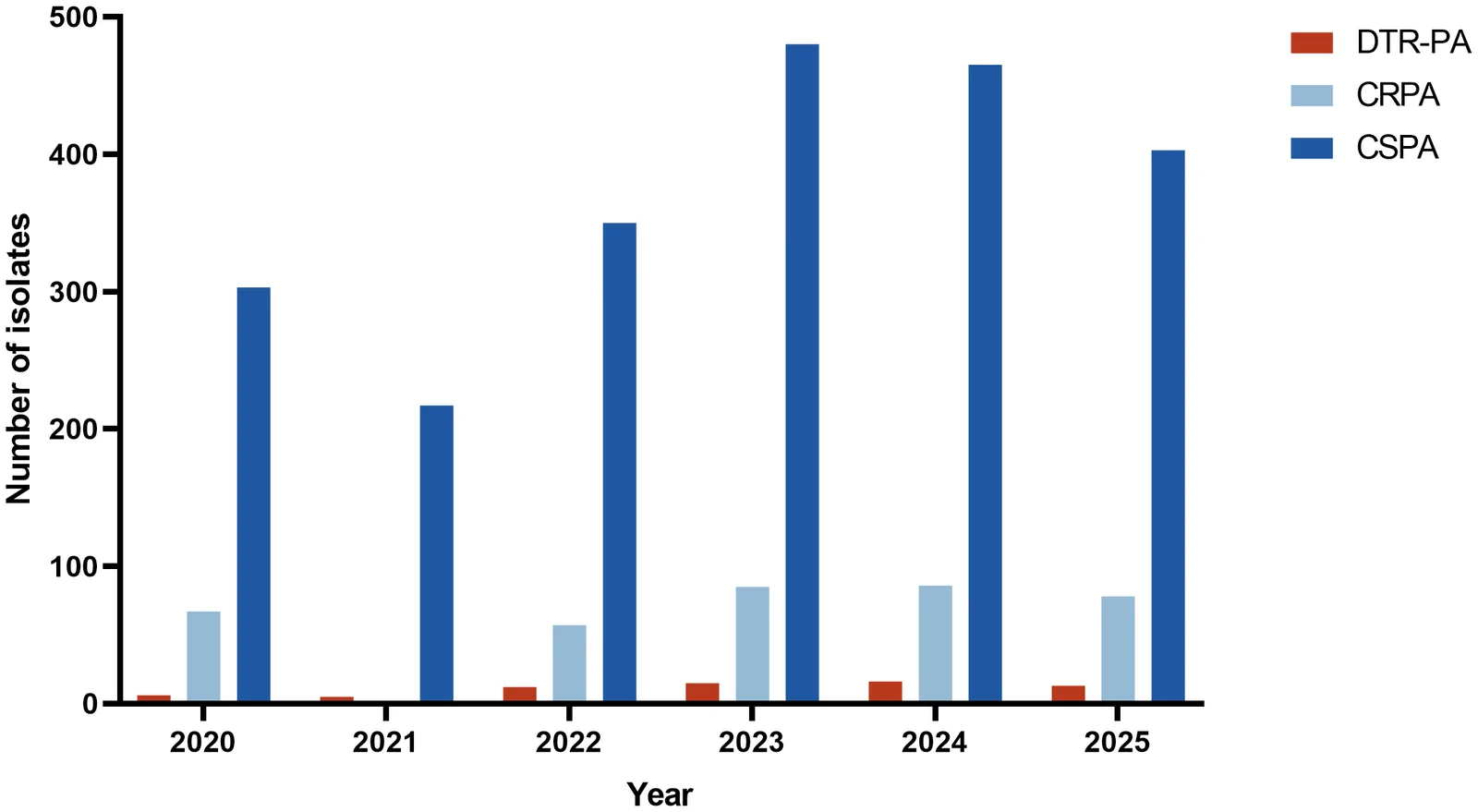
Annual distribution of DTR-PA, CRPA, and CSPA isolates from 2020 to 2025. CSPA, carbapenem-susceptible *Pseudomonas aeruginosa;* CRPA, carbapenem-resistant *P. aeruginosa*; DTR-PA, difficult-to-treat resistant *P. aeruginosa*.

We further analyzed the evolution of resistance patterns among DTR-PA isolates. Among the 67 DTR-PA cases, 67% were identified as DTR-PA at the first isolation. Additionally, 25% of patients had a prior history of CRPA isolation before progression to DTR-PA, while 8% of patients were first identified as CSPA and subsequently evolved from CSPA to CRPA and ultimately to DTR-PA. The detailed transition patterns are shown in **Figure 3**.

**Figure 3:**
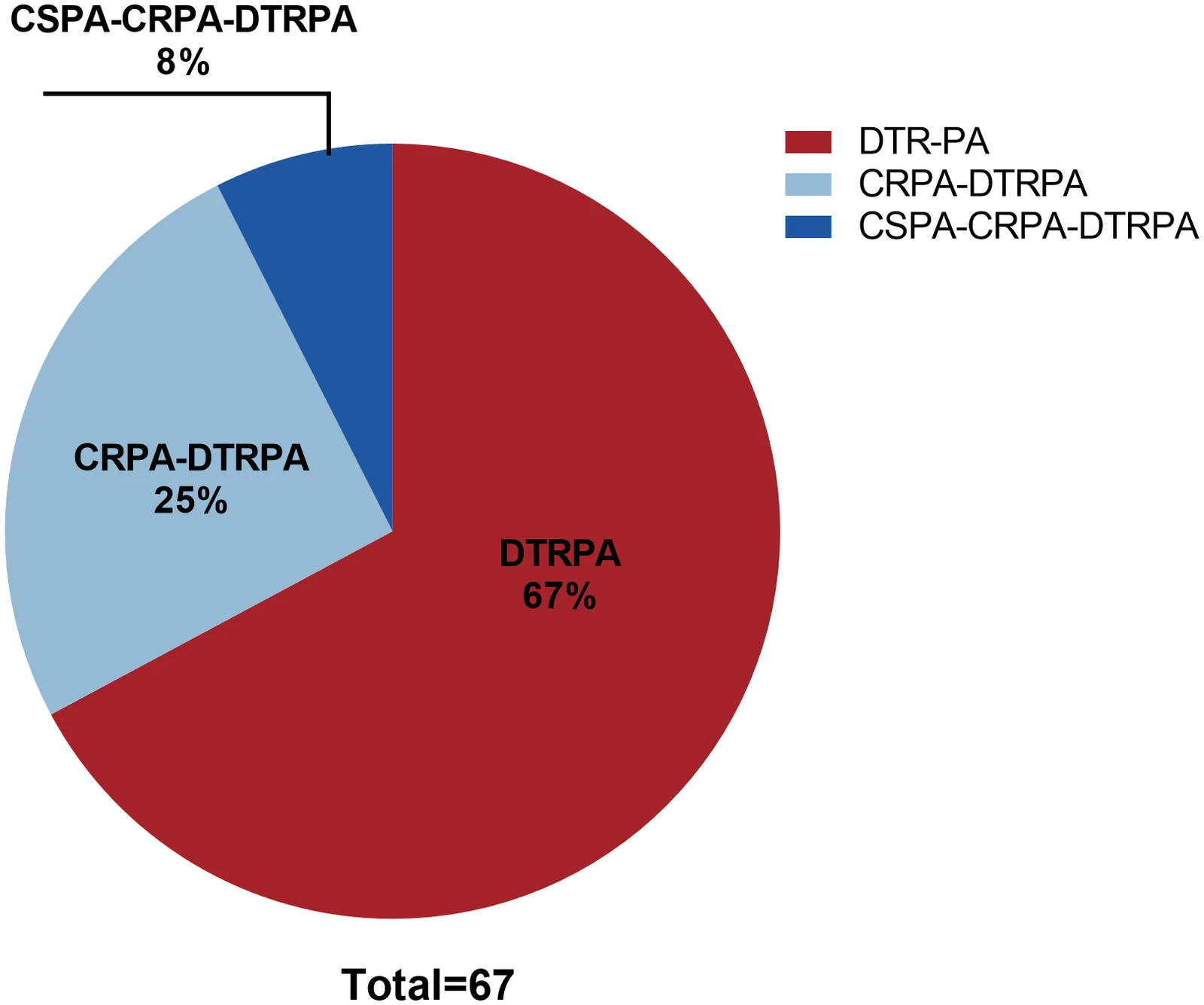
Antimicrobial resistance trends of clinically isolated DTR-PA in our hospital. CSPA, carbapenem-susceptible *Pseudomonas aeruginosa;* CRPA, carbapenem-resistant *P. aeruginosa*; DTR-PA, difficult-to-treat resistant *P. aeruginosa*.

We also compared the susceptibility profiles of the three phenotypic groups against eight commonly used first-line antipseudomonal agents. DTR-PA showed non-susceptibility to all eight tested antipseudomonal agents. For CRPA, non-susceptibility rates to meropenem or imipenem ranged from 90.17% to 91.40%. In contrast, CSPA showed overall low non-susceptibility rates, with only aztreonam exceeding 25%. The detailed non-susceptibility profiles are presented in **Figure 4**.

**Figure 4:**
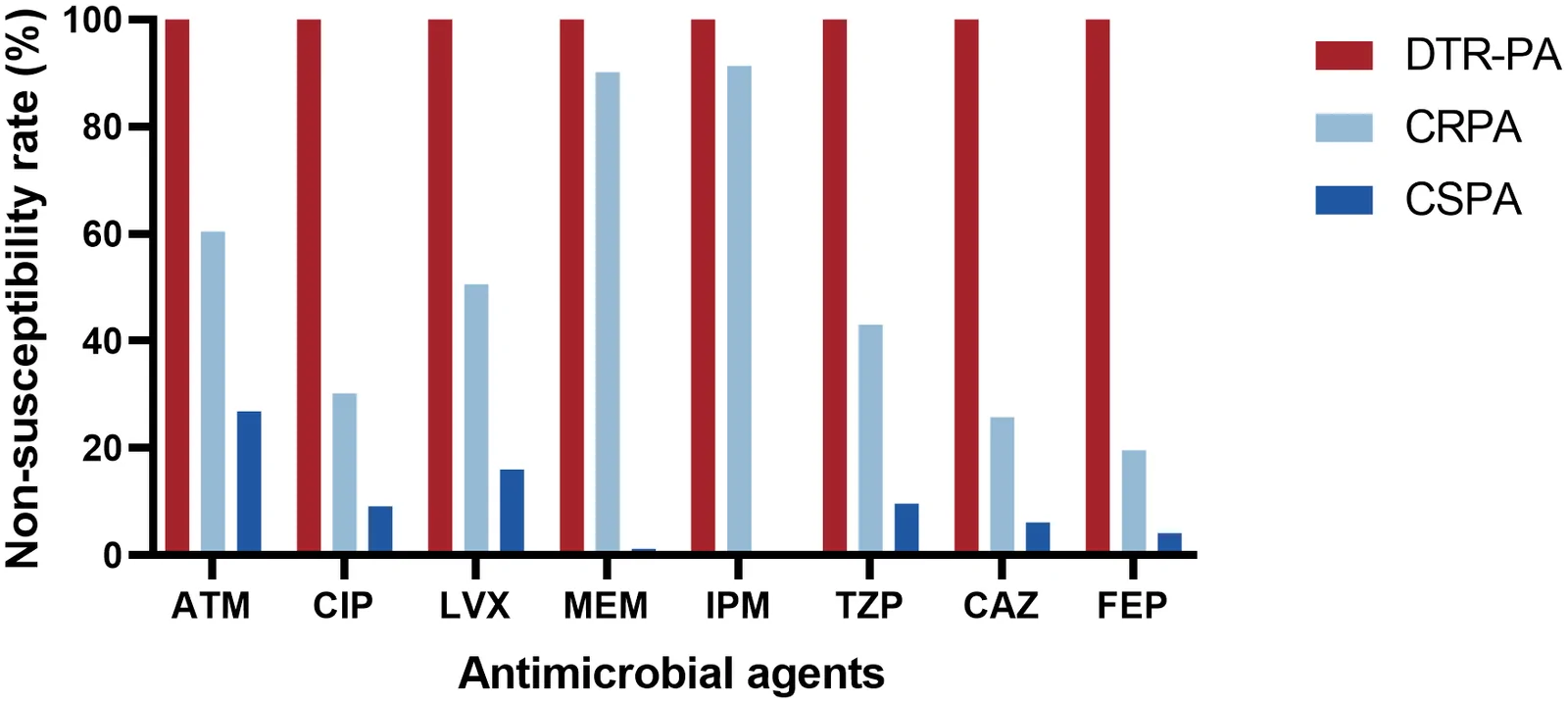
Non-susceptibility rates of CSPA, CRPA, and DTR-PA clinical isolates to eight antimicrobial agents. This figure illustrates the rates of non-susceptibility to eight first-line antibiotics among clinical isolates of *P. aeruginosa*, reflecting their intermediate and resistance profiles according to the CLSI interpretive criteria. CSPA, carbapenem-susceptible *Pseudomonas aeruginosa;* CRPA, carbapenem-resistant *P. aeruginosa*; DTR-PA, difficult-to-treat resistant *P. aeruginosa*; ATM, Aztreonam; CIP, Ciprofloxacin; LVX, Levofloxacin; MEM, Meropenem; IPM, Imipenem; TZP, Piperacillin/Tazobactam; CAZ, Ceftazidime; FEP, Cefepime.

### 3.2 Clinical characteristics of *P. aeruginosa* isolates with different resistance phenotypes

A total of 335 patients with *P. aeruginosa* infection were included, comprising 67 DTR-PA cases, 134 CRPA cases, and 134 CSPA cases. No significant differences were observed in age or sex distribution among the three groups. However, significant differences were identified in department distribution, specimen type, hospitalization characteristics, prior healthcare exposure, and infection-related factors (**Table 1**).

**Table 1:** Clinical characteristics of patients in different cohorts.

Characteristic	Total (n=335)	CSPA (n=134)	CRPA (n=134)	DTR-PA (n=67)	*p*
Demographic and clinical characteristics					
Demographics					
Gender, male, n (%)	232 (69.3%)	84 (62.7%)	96 (71.6%)	52 (77.6%)	0.072
Age (years), mean (SD)	62.84 (15.58)	62.08 (15.07)	63.85 (16.85)	62.34 (13.96)	0.623
BMI (kg/m^2^), mean (SD)	22.69 (4.64)	23.57 (5.05)	22.35 (4.27)	21.56 (4.17)	0.010
Department, n (%)					<0.001
ICU	103 (30%)	26 (19.4%)	52 (38.8%)	25 (37.3%)	
Respiratory Medicine	52 (15.5%)	30 (22.4%)	12 (9.0%)	10 (14.9%)	
Hematology	30 (9.0%)	9 (6.7%)	16 (11.9%)	5 (7.5%)	
Neurosurgery	17 (5.1%)	1 (0.7%)	12 (9.0%)	4 (6.0%)	
Infectious Diseases	15 (4.5%)	6 (4.5%)	7 (5.2%)	2 (3.0%)	
Rheumatology	12 (3.6%)	7 (5.2%)	4 (3.0%)	1 (1.5%)	
Endocrinology	12 (3.6%)	4 (3.0%)	0 (0.0%)	8 (11.9%)	
Neurology	12 (3.6%)	4 (3.0%)	7 (5.2%)	1 (1.5%)	
Geriatrics	11 (3.3%)	7 (5.2%)	2 (1.5%)	2 (3.0%)	
Other	71 (21.2%)	40 (29.9%)	22 (16.4%)	9 (13.4%)	
Hospital characteristics					
Times of Hospitalizations, median (IQR)	1.00 (1.00, 3.00)	1.00 (1.00, 2.00)	1.00 (1.00, 2.00)	2.00 (1.00, 3.50)	0.072
Host factors and prior medication exposure					
Special drug exposure, n (%)					
Corticosteroid	80 (23.9%)	40 (29.9%)	31 (23.1%)	9 (13.4%)	0.035
Immunosuppressant	10 (3.0%)	2 (1.5%)	6 (4.5%)	2 (3.0%)	0.357
SGLT2 inhibitor	9 (2.7%)	4 (3.0%)	1 (0.7%)	4 (6.0%)	0.094
Advanced antibiotics	186 (55.5%)	74 (55.2%)	80 (59.7%)	32 (47.8%)	0.274
Underlying medical conditions, n (%)					
COPD/Bronchiectasis	38 (11.3%)	18 (13.4%)	10 (7.5%)	10 (14.9%)	0.179
Diabetes	62 (18.5%)	30 (22.4%)	12 (9.0%)	20 (29.9%)	0.001
Cardiovascular	107 (31.9%)	51 (38.1%)	32 (23.9%)	24 (35.8%)	0.034
Chronic kidney disease	24 (7.2%)	14 (10.4%)	9 (6.7%)	1 (1.5%)	0.065
Tumor	78 (23.3%)	29 (21.6%)	37 (27.6%)	12 (17.9%)	0.260
Rheumatologic diseases	5 (1.5%)	1 (0.7%)	4 (3.0%)	0 (0.0%)	0.169
Cerebrovascular	56 (16.7%)	10 (7.5%)	26 (19.4%)	20 (29.9%)	<0.001
Rheumatic autoimmune	24 (7.2%)	12 (9.0%)	10 (7.5%)	2 (3.0%)	0.298
Trauma	44 (13.1%)	14 (10.4%)	23 (17.2%)	7 (10.4%)	0.204
Healthcare exposure and disease severity					
Disease severity, n (%)					
Critically ill	216 (64.5%)	59 (44.0%)	107 (79.9%)	50 (74.6%)	<0.001
ICU admission during the current hospitalization	139 (41.5%)	32 (23.9%)	75 (56.0%)	32 (47.8%)	<0.001
Prior healthcare exposure, n (%)					
Hospitalization within the previous 3 months	214 (63.9%)	88 (65.7%)	80 (59.7%)	46 (68.7%)	0.394
ICU stay within the previous 3 months	50 (14.9%)	13 (9.7%)	14 (10.4%)	23 (34.3%)	<0.001
3-month antibiotic exposure history	162 (48.4%)	60 (44.8%)	58 (43.3%)	44 (65.7%)	0.006
Healthcare utilization					
Length of hospital stay (days), median (IQR)	17.00 (10.00, 29.00)	14.00 (8.00, 21.50)	24.00 (13.00, 37.00)	18.00 (11.00, 23.50)	<0.001
Medication costs (CNY), mean (SD)	29073.86 (47869.95)	14460.22 (27879.67)	37026.50 (39431.14)	42395.86 (78452.95)	<0.001
Antibiotic costs (CNY), median (IQR)	2867.81 (751.42, 9343.31)	1236.24 (314.50, 3212.81)	5497.74 (1354.98, 12521.29)	4180.62 (1792.52, 12664.98)	<0.001
Proportion of antibiotic costs, median (IQR)	0.26 (0.14, 0.46)	0.24 (0.11, 0.49)	0.23 (0.12, 0.42)	0.33 (0.20, 0.48)	0.092
Invasive procedures, n (%)	166 (49.6%)	24 (17.9%)	92 (68.7%)	50 (74.6%)	<0.001
Endotracheal intubation or tracheostomy	121 (36.1%)	20 (14.9%)	64 (47.8%)	37 (55.2%)	<0.001
Central venous catheterization	98 (29.3%)	19 (14.2%)	47 (35.1%)	32 (47.8%)	<0.001
Urinary catheter	77 (23.0%)	17 (12.7%)	35 (26.1%)	25 (37.3%)	<0.001
Chest drainage	49 (14.6%)	10 (7.5%)	24 (17.9%)	15 (22.4%)	0.007
Lumbar puncture/Bone marrow aspiration	16 (4.8%)	4 (3.0%)	4 (3.0%)	8 (11.9%)	0.009
Laboratory parameters					
Liver function, mean (SD)					
ALT (U/L)	39.17 (106.43)	42.89 (160.37)	39.53 (43.04)	30.89 (37.08)	0.759
AST (U/L)	43.75 (85.77)	47.14 (124.27)	43.34 (43.70)	37.68 (45.14)	0.767
TBIL (μmol/L)	17.04 (17.25)	14.73 (13.61)	20.80 (20.82)	14.18 (14.68)	0.006
TP (g/L)	60.29 (10.53)	62.90 (9.61)	57.99 (11.24)	59.63 (9.69)	0.001
ALB (g/L)	32.52 (6.46)	34.62 (6.67)	31.19 (6.35)	31.00 (5.06)	<0.001
ALP (U/L)	101.80 (63.28)	98.24 (42.32)	101.90 (76.30)	109.38 (71.45)	0.628
GGT (U/L)	60.98 (91.99)	62.38 (83.93)	66.04 (112.35)	46.70 (49.88)	0.504
Renal function, mean (SD)					
Ccr (mL/min)	112.22 (166.83)	95.29 (49.50)	118.25 (214.18)	129.30 (189.16)	0.388
Inflammatory markers					
Peak WBC(×10^9^/L), median (IQR)	11.32 (7.42, 17.06)	9.00 (6.28, 13.13)	13.06 (8.80, 19.01)	14.64 (10.39, 19.15)	<0.001
Peak neutrophil percentage (%), mean (SD)	81.57 (16.63)	76.29 (18.61)	85.54 (14.36)	85.63 (11.92)	<0.001
Peak CRP (mg/L), median (IQR)	58.58 (15.83, 143.22)	25.95 (8.67, 94.86)	85.29 (25.40, 154.54)	99.00 (32.06, 188.08)	<0.001
Peak PCT (ng/mL), median (IQR)	1.41 (0.46, 7.34)	0.64 (0.35, 2.18)	2.03 (0.66, 9.68)	1.58 (0.56, 6.58)	<0.001
Peak IL-6 (pg/mL), median (IQR)	88.42 (30.32, 430.25)	38.50 (12.72, 256.26)	127.00 (46.25, 512.00)	123.00 (55.80, 406.75)	0.008
Peak ESR (mm/h), median (IQR)	35.90 (14.00, 79.50)	27.00 (12.00, 71.00)	43.50 (18.25, 82.25)	38.00 (16.00, 79.25)	0.291
Infection-related characteristics					
Site of infection, n(%)					
Pulmonary	240 (71.6%)	85 (63.4%)	105 (78.4%)	50 (74.6%)	0.021
Bloodstream	20 (6.0%)	7 (5.2%)	10 (7.5%)	3 (4.5%)	0.628
Urinary tract infection	18 (5.4%)	9 (6.7%)	3 (2.2%)	6 (9.0%)	0.093
Skin and soft tissue	20 (6.0%)	6 (4.5%)	3 (2.2%)	11 (16.4%)	<0.001
Central nervous system	5 (1.5%)	1 (0.7%)	3 (2.2%)	1 (1.5%)	0.602
Intra-abdominal	13 (3.9%)	3 (2.2%)	6 (4.5%)	4 (6.0%)	0.390
Non-bacterial co-infection, n (%)					
Virus	37 (11.0%)	15 (11.2%)	17 (12.7%)	5 (7.5%)	0.536
Fungi	22 (6.6%)	4 (3.0%)	13 (9.7%)	5 (7.5%)	0.081
Microbiological characteristics					
Bacterial co-isolates, n (%)					
*Acinetobacter baumannii*	55 (16.4%)	8 (6.0%)	28 (20.9%)	19 (28.4%)	<0.001
*Klebsiella pneumoniae*	52 (15.5%)	13 (9.7%)	20 (14.9%)	19 (28.4%)	0.003
*Stenotrophomonas maltophilia*	16 (4.8%)	4 (3.0%)	9 (6.7%)	3 (4.5%)	0.356
*Escherichia coli*	13 (3.9%)	5 (3.7%)	6 (4.5%)	2 (3.0%)	0.869
*Enterobacter cloacae*	12 (3.6%)	4 (3.0%)	7 (5.2%)	1 (1.5%)	0.362
*Corynebacterium striatum*	16 (4.8%)	4 (3.0%)	7 (5.2%)	5 (7.5%)	0.356
*Staphylococcus aureus*	9 (2.7%)	1 (0.7%)	6 (4.5%)	2 (3.0%)	0.166
Specimen type, n (%)					0.018
Sputum	246 (73.4%)	99 (73.9%)	101 (75.4%)	46 (68.7%)	
Blood	8 (2.4%)	6 (4.5%)	2 (1.5%)	0 (0.0%)	
Bronchoalveolar lavage fluid,	14 (4.2%)	3 (2.2%)	6 (4.5%)	5 (7.5%)	
Urine	15 (4.5%)	9 (6.7%)	6 (4.5%)	0 (0.0%)	
Secretion	25 (7.5%)	7 (5.2%)	7 (5.2%)	11 (16.4%)	
Other	27 (8.1%)	10 (7.5%)	12 (9.0%)	5 (7.5%)	

Data are presented as mean (SD), median (IQR), or n (%), as appropriate. Between-group comparisons were performed using one-way analysis of variance, the Kruskal–Wallis test, or Pearson’s chi-square test, as appropriate. CSPA, carbapenem-susceptible *Pseudomonas aeruginosa;* CRPA, carbapenem-resistant *P. aeruginosa*; DTR-PA, difficult-to-treat resistant *P. aeruginosa*; BMI, body mass index; ICU, intensive care unit; COPD, chronic obstructive pulmonary disease; SGLT2, sodium-glucose cotransporter 2; ALT, alanine aminotransferase; AST, aspartate aminotransferase; TBIL, total bilirubin; TP, total protein; ALB, albumin; ALP, alkaline phosphatase; GGT, gamma-glutamyl transferase; Ccr, creatinine clearance rate; WBC, white blood cell count; CRP, C-reactive protein; PCT, procalcitonin; IL-6, interleukin-6; ESR, erythrocyte sedimentation rate; SD, standard deviation; IQR, interquartile range; CNY, Chinese yuan.

Compared with the CSPA group, both CRPA and DTR-PA groups exhibited substantially greater clinical severity, including higher rates of critical illness, ICU admission during the current hospitalization, invasive procedures, longer hospital stay, and increased antibiotic-related expenditures (all *p* < 0.05).

A progressive increase in healthcare-associated exposures was observed across the resistance spectrum from CSPA to CRPA and DTR-PA. Specifically, the prevalence of endotracheal intubation or tracheostomy, central venous catheterization (CVC), chest drainage, prior ICU stay, and prior antibiotic exposure increased substantially with escalating resistance phenotype. Notably, markers of systemic inflammation also demonstrated a stepwise upward trend, with increasing peak white blood cell counts, neutrophil percentages, and inflammatory biomarker levels across the three resistance phenotypes, indicating a progressively heightened inflammatory burden accompanying resistance escalation. In addition, DTR-PA infections were more frequently associated with co-infection by other bacterial pathogens, particularly *Acinetobacter baumannii* and *Klebsiella pneumoniae*, as well as a higher prevalence of diabetes and cerebrovascular disease.

Overall, the findings demonstrated a stepwise increase in clinical complexity, inflammatory burden, and disease severity across CSPA, CRPA, and DTR-PA phenotypes.

### 3.3 Risk factors across the antimicrobial resistance gradient in *P. aeruginosa*

#### 3.3.1 Independent risk factors for DTR-PA infection

Univariable analysis identified multiple factors associated with DTR-PA infection (**Supplementary Table 1**). Variables selected by LASSO regression were subsequently entered into a multivariable Firth logistic regression model. As shown in **Table 2**, antibiotic use within the previous 3 months (OR 3.468, 95% CI 1.825-6.867; *p* < 0.001), cerebrovascular disease (OR 2.898, 95% CI 1.400-5.935; *p* = 0.005), CVC (OR 2.757, 95% CI 1.350-5.693; *p* = 0.005), lumbar puncture or bone marrow aspiration (OR 4.325, 95% CI 1.244-14.684; *p* = 0.022), endotracheal intubation or tracheostomy (OR 2.292, 95% CI 1.138-4.645; *p* = 0.020), and diabetes (OR 2.464, 95% CI 1.165-5.123; *p* = 0.019) were independently associated with DTR-PA infection.

**Table 2:** DTR-PA vs non-DTR-PA, selection followed by multivariable Firth logistic regression.

Variable	OR	Lower	Upper	*p*
3-month antibiotic exposure history	3.468	1.825	6.867	<0.001
Cerebrovascular Disease	2.898	1.400	5.935	0.005
CVC	2.757	1.350	5.693	0.005
Lumbar Puncture or Bone Marrow Aspiration	4.325	1.244	14.684	0.022
Endotracheal intubation or tracheostomy	2.292	1.138	4.645	0.020
Diabetes	2.464	1.165	5.123	0.019

DTR-PA, difficult-to-treat resistant *Pseudomonas aeruginosa*; CVC, Central venous catheterization.

#### 3.3.2 Risk factors associated with progression from CRPA to DTR-PA

Univariable analysis identified multiple factors associated with DTR-PA infection (**Supplementary Table 2**). Variables selected by LASSO regression were subsequently entered into a multivariable Firth logistic regression model. As shown in **Table 3**, compared with CRPA infection, diabetes (OR 6.955, 95% CI 2.991-16.928; *p* < 0.001), ICU stay within the previous 3 months (OR 4.665, 95% CI 1.962-11.517; *p* < 0.001), lumbar puncture or bone marrow aspiration (OR 8.317, 95% CI 2.340-33.096; *p* < 0.001), and chronic obstructive pulmonary disease (COPD) or bronchiectasis (OR 3.724, 95% CI 1.350-10.386; *p* = 0.012) were independently associated with DTR-PA infection, indicating an increased likelihood of progression to the higher-level resistance phenotype.

**Table 3:** DTR-PA vs CRPA, LASSO selection followed by multivariable Firth logistic regression.

Variable	OR	Lower	Upper	*p*
Diabetes	6.955	2.991	16.928	<0.001
ICU stay within the previous 3 months	4.665	1.962	11.517	0.001
Lumbar Puncture or Bone Marrow Aspiration	8.317	2.340	33.096	0.001
3-month antibiotic exposure history	1.623	0.787	3.373	0.190
COPD or Bronchiectasis	3.724	1.350	10.386	0.012

DTR-PA, difficult-to-treat resistant *Pseudomonas aeruginosa*; CRPA, carbapenem-resistant *P. aeruginosa*; ICU, intensive care unit; COPD, chronic obstructive pulmonary disease.

#### 3.3.3 Risk factors associated with acquisition of antimicrobial resistance in *P. aeruginosa*

Univariable analysis identified multiple factors associated with resistant *P. aeruginosa* infection (**Supplementary Table 3**). Variables selected by LASSO regression were subsequently entered into a multivariable Firth logistic regression model. As shown in **Table 4**, co-infection with other bacteria (OR 1.818, 95% CI 1.040-3.176; *p* = 0.036), endotracheal intubation or tracheostomy (OR 2.499, 95% CI 1.217-5.134; *p* = 0.013), and prolonged hospitalization (14–90 days) (OR 2.047, 95% CI 1.162-3.605; *p* = 0.013) were independently associated with resistant *P. aeruginosa* infection, whereas higher albumin (ALB; OR 0.955, 95% CI 0.914-0.997; *p* = 0.037) and BMI (OR 0.920, 95% CI 0.863-0.980; *p* = 0.010) were independently associated with modestly lower odds of resistant *P. aeruginosa* infection on a per-unit basis. .

**Table 4:** R-PA vs CSPA, LASSO selection followed by multivariable Firth logistic regression.

Variable	OR	Lower	Upper	*p*
ALB	0.955	0.914	0.997	0.037
Co-infection with Other Bacteria	1.818	1.040	3.176	0.036
Critically ill	1.498	0.816	2.752	0.192
Endotracheal intubation or tracheostomy	2.499	1.217	5.134	0.013
BMI	0.920	0.863	0.980	0.010
Cerebrovascular Disease	2.169	0.967	4.863	0.060
CVC	1.419	0.699	2.882	0.333
Length of stay, 14–90 days	2.047	1.162	3.605	0.013
Length of stay, >90 days	2.612	0.249	27.390	0.423

CSPA, carbapenem-susceptible *Pseudomonas aeruginosa*; R-PA, resistant *P. aeruginosa* (including CRPA and DTR-PA); ALB, albumin; BMI, body mass index; CVC, Central venous catheterization.

### 3.4 Model performance and sensitivity analysis

The performance evaluation results for all three models are summarized in **Table 5**. All three models demonstrated satisfactory discrimination and calibration performance. The area under the curve (AUC) was 0.815 for the DTR-PA vs. non-DTR-PA model, 0.779 for the DTR-PA vs. CRPA model, and 0.806 for the R-PA vs. CSPA model, indicating good discriminative ability. The corresponding Nagelkerke R² values were 0.293, 0.303, and 0.329, respectively, suggesting moderate explanatory power. Sensitivity analyses showed complete agreement between the multiple imputation (MI) and complete-case analysis (CCA) results across all three models (100% concordance), supporting the robustness of the findings.

**Table 5:** Performance evaluation of three multivariate regression models.

Indicators	DTR-PA vs non-DTR-PA	DTR-PA vs CRPA	R-PA vs CSPA
AUC	0.815	0.779	0.806
Nagelkerke R^2^	0.293	0.303	0.329
Consistency Rate (MI-CCA)	100%	100%	100%

AUC, area under the curve, Consistency rate (MI–CCA) was defined as the proportion of variables showing consistent significance and direction of association between multiple imputation and complete-case analysis. DTR-PA, difficult-to-treat resistant *Pseudomonas aeruginosa*; CRPA, carbapenem-resistant *P. aeruginosa*; R-PA, resistant *P. aeruginosa* (including CRPA and DTR-PA.).

## 4 Discussion

In this six-year observational study, we characterized a clinically relevant resistance continuum spanning CSPA, CRPA, and DTR-PA and demonstrated that increasing levels of antimicrobial resistance were associated with greater exposure to critical illness, heightened systemic inflammation, and progressive deterioration in nutritional and immune status. These findings provide clinical evidence supporting a stepwise progression of antimicrobial resistance in *P. aeruginosa* and are consistent with the findings of Gómez-Zorrilla et al. (2017), who reported that infections caused by extensively drug-resistant *P. aeruginosa* were associated with significantly stronger inflammatory responses and more severe clinical manifestations than infections caused by susceptible isolates.

Antimicrobial susceptibility results in this study were interpreted according to the CLSI M100 criteria. In 2019, EUCAST revised the definitions of the S, I, and R categories, redefining I as “susceptible, increased exposure,” whereas S denotes “susceptible, standard dosing regimen” (Nabal Díaz et al., 2022). Thus, under the EUCAST framework, both S and I are considered susceptible categories, although different levels of antimicrobial exposure are required. The DTR definition proposed by Kadri et al. classified isolates as DTR when they showed intermediate or resistant results to all relevant first-line β-lactams, including carbapenems, and fluoroquinolones (Kadri et al., 2018). Therefore, applying current EUCAST breakpoints and category definitions could alter the classification of DTR-PA, because isolates categorized as I under EUCAST should not be interpreted as non-susceptible. In addition, numerical breakpoint differences between CLSI and EUCAST may further affect susceptibility categorization. Because the original MIC or inhibition-zone data were not reinterpreted using EUCAST criteria, the direction and magnitude of any resulting reclassification could not be determined. Accordingly, the DTR-PA classification and related findings in this study should be interpreted within the CLSI framework.

ICU settings appeared to be an important reservoir for multidrug-resistant organisms and a major healthcare environment associated with resistant *P. aeruginosa* infections, and the rates of various invasive procedures had risen significantly. Specifically, we observed a progressive increase in the frequency of invasive procedures across the resistance continuum, including airway interventions, central venous catheterization, and urinary catheterization. Invasive medical devices disrupt normal host barriers and provide favorable surfaces for biofilm formation by *P. aeruginosa*. Biofilm-associated bacteria exhibit enhanced tolerance to antimicrobial agents and host immune defenses, facilitating persistent colonization and the selection of resistant subpopulations (Itani et al., 2025b). Inflammatory markers exhibited a gradient variation across groups, with peak C-reactive protein (CRP) levels rising from 25.95 (mg/L) in the CSPA group to 99.00 (mg/L) in the DTR-PA group, suggesting that the higher the level of resistance, the greater the inflammatory burden associated with systemic infection. Mixed infections involving multiple bacterial species were another characteristic of this gradient; the proportion of *Acinetobacter baumannii* infections in the DTR-PA group reached 28.4%, far higher than in the CSPA group (6.0%) and the CRPA group (20.9%). Polymicrobial infections often necessitate broad-spectrum or combination antimicrobial therapy, thereby increasing antimicrobial selective pressure and facilitating resistance evolution. Based on the clinical characteristics observed in the resistance gradient described above, we further investigated risk factors that may influence the progression of *P. aeruginosa* resistance.

Hospitalization itself is a well-recognized risk factor for infections caused by resistant organisms, as prolonged hospital stays increase opportunities for exposure to healthcare-associated pathogens. This effect is particularly pronounced in ICU settings, where patients are more likely to undergo invasive procedures and receive broad-spectrum antimicrobial therapy, thereby facilitating the selection, colonization, and persistence of resistant bacteria (Harris et al., 2011; Van der Bij et al., 2011; Voor in ’t Holt et al., 2014; Yuan et al., 2023). The inverse associations of higher BMI and ALB levels with R-PA infection should be interpreted cautiously, as the corresponding ORs were close to 1, indicating relatively modest associations on a per-unit basis rather than strong protective effects. These findings should therefore be interpreted cautiously and should not be considered evidence of a causal protective effect. Hypoalbuminemia may reflect both poor nutritional status and ongoing systemic inflammation, which can impair innate immune responses, reduce neutrophil chemotaxis and bactericidal activity, and compromise pathogen clearance. These alterations may contribute to a vicious cycle of infection, immune dysfunction, and persistent colonization by resistant organisms. Conversely, adequate nutritional status may enhance host defenses and reduce susceptibility to resistant infections. ALB has long been recognized as both a nutritional risk indicator and an inflammatory biomarker, and previous studies have demonstrated that improved ALB levels are significantly associated with successful antimicrobial treatment outcomes (Li et al., 2025). Furthermore, research findings show that an appropriate BMI may be associated with more favorable infection-related outcomes, possibly reflecting better nutritional status and immune function.

In the DTR-PA vs. CRPA model, diabetes and COPD or bronchiectasis were independent risk factors for the progression of CRPA to DTR-PA. This suggests that the progression from CRPA to DTR-PA may be more closely associated with exposure to chronic underlying diseases. Unlike factors associated with the initial acquisition of resistance, these findings suggest that resistance escalation from CRPA to DTR-PA may be driven more strongly by chronic host-related factors and repeated healthcare exposure. Previous studies have identified diabetes as a risk factor for CRPA infection (Hu et al., 2021; Zhang et al., 2025), however, its role in the progression from CRPA to DTR-PA remains poorly understood. In this study, diabetes was the strongest risk factor for the progression of antibiotic resistance (OR = 6.955), with a risk magnitude significantly higher than that of most clinical factors. Persistent hyperglycemia impairs innate immune defenses, including neutrophil chemotaxis, phagocytosis, and bactericidal activity, thereby facilitating chronic colonization and recurrent infection (Darwitz Benjamin et al., 2024). Additionally, patients with diabetes are more prone to skin and soft tissue infections as well as diabetic foot ulcers (Casqueiro et al., 2012), and repeated hospitalizations and frequent use of broad-spectrum anti-Pseudomonas antibiotics continuously exert selective pressure, inducing the emergence of multidrug-resistant bacteria. Patients with COPD or bronchiectasis are prone to chronic infections and airway inflammation due to impaired airway structure. At the same time, factors such as primary ciliary dyskinesia, mucus retention, and impaired ciliary clearance provide a favorable environment for *P. aeruginosa* colonization (Reynolds and Kollef, 2021). During acute exacerbations, these patients often require repeated hospitalizations and antimicrobial therapy, making them more susceptible to sustained selection pressure for resistance (Chen et al., 2020) and more prone to DTR-PA infections.

The first two models examined risk factors associated with the evolution of *P. aeruginosa* resistance at two levels: the onset of resistance (R-PA vs. CSPA) and the progression of resistance (DTR-PA vs. CRPA). From a clinical perspective, the more critical issue lies in the early identification of high-risk populations for DTR-PA. Therefore, this study further developed a comparative model between DTR-PA and non-DTR-PA to identify independent risk factors with clinical predictive value, thereby providing a basis for the early identification and intervention of high-risk patients.

Compared with the first two models, the DTR-PA versus the non-DTR-PA model better reflects the clinical characteristics most relevant to the early identification of patients at risk for DTR-PA infection. The results showed that a history of antimicrobial use within the past 3 months, diabetes, cerebrovascular disease, central venous catheterization, tracheostomy, and lumbar puncture or bone marrow aspiration were all independent risk factors. Compared with the first two models, this model reflects the high-risk characteristics with the greatest clinical value for identifying DTR-PA during its development. These risk factors can be broadly categorized into three domains: antimicrobial exposure, host-related comorbidities, and invasive healthcare interventions. Unlike the first two models, a history of antimicrobial use within the past 3 months and cerebrovascular disease were retained only in this model, indicating that these two factors may have greater clinical predictive value for DTR-PA infections. Antibiotic exposure within the previous 3 months was independently associated with DTR-PA infection. Antimicrobial exposure is a major risk factor for multidrug-resistant *P. aeruginosa* (MDR-PA) infections in hospitalized patients; antimicrobial pressure can significantly increase the risk of acquiring multidrug-resistant strains (Raman et al., 2018; Chen et al., 2020).

Repeated exposure to broad-spectrum antimicrobial agents may promote the enrichment of resistant subpopulations under sustained selective pressure and induce bacteria to gradually acquire higher levels of resistance through mechanisms such as AmpC, β-lactamase overexpression, OprD pore protein deletion, and high expression of efflux pumps (Pang et al., 2019). The results of this study further confirm that recent exposure to antimicrobial agents is closely associated with the occurrence of DTR-PA, suggesting that the rational use of antimicrobial agents remains a key factor in preventing the development of DTR-PA. In addition, patients with cerebrovascular diseases, such as stroke, often experience prolonged bed rest and swallowing dysfunction, which significantly increases the risk of pulmonary infections (Martino et al., 2005). Consequently, patients with cerebrovascular diseases may be more likely to receive broad-spectrum antimicrobial therapy due to recurrent infections and prolonged exposure to healthcare settings, thereby increasing the risk of resistance. Furthermore, diabetes, endotracheal intubation/tracheostomy, and bone marrow aspiration/lumbar puncture recurred in all three models, suggesting that these factors may be present throughout the entire process of *P. aeruginosa* resistance development and progression to DTR-PA.

Based on the results of our study using the three models described above, we found that a history of antimicrobial use within the past 3 months is a significant risk factor for DTR-PA infection. Under sustained selective pressure from antimicrobial agents, *P. aeruginosa* may develop resistance to β-lactams, carbapenems, and fluoroquinolones through the interaction of multiple resistance mechanisms—including reduced OprD expression, upregulation of MexAB-OprM, and overexpression of AmpC enzymes—gradually leading to the emergence of DTR-PA (Tamma et al., 2024). Long-term medical exposure, such as invasive procedures like tracheostomy and central venous catheterization, as well as a history of ICU admission within the past three months, may all contribute to the development of DTR-PA. Patients undergoing endotracheal intubation or tracheostomy are more susceptible to persistent colonization and infection by *Pseudomonas aeruginosa* due to compromised natural airway barriers, reduced clearance of respiratory secretions, and biofilm formation on the surface of artificial airways. At the same time, such critically ill patients often require prolonged mechanical ventilation and broad-spectrum antimicrobial therapy. Under continuous selective pressure from antimicrobial agents, resistant strains are more easily selected and gradually gain a dominant position. Mechanical ventilation-related equipment and respiratory medical devices may also serve as important vectors for the transmission of resistant bacteria, further increasing the risk of resistant *P. aeruginosa* infections (Qin et al., 2022; He et al., 2023; Xiao et al., 2025).

This study identified risk factors for DTR-PA infection, which is of great significance for clinical prevention and control. First, the risk factors we have identified will help clinicians proactively identify hospitalized patients at high risk for DTR-PA infection and implement timely interventions, such as proactive pathogen testing. At the same time, attention must be paid to the rational use of antimicrobial agents; clinicians should strictly adhere to the indications for broad-spectrum antibiotics, avoid prolonged or repeated use of the same class of antibiotics, and promptly implement step-down therapy based on laboratory results to reduce selection pressure for resistance. Furthermore, since the ICU is the primary site of infection for drug-resistant *P. aeruginosa*, this highlights the need for healthcare institutions to strengthen environmental disinfection, ensure the proper use of medical devices, standardize the management of invasive procedures, and enforce personal hygiene among healthcare workers. These measures are crucial for preventing the spread of drug-resistant bacteria and reducing DTR-PA infections.

### 4.1 Limitations

This study has several limitations. First, this was a single-center study conducted in a tertiary teaching hospital, which may limit the generalizability of the findings to other healthcare settings and regions.

Second, the retrospective study design is inherently subject to selection and information bias. Some patients were excluded because complete antimicrobial susceptibility testing results were unavailable, which may have introduced selection bias during patient enrollment. In addition, patients were classified according to the highest resistance phenotype documented during the study period rather than according to the phenotype of the first eligible *P. aeruginosa* isolate. This strategy enabled the identification of patients who subsequently developed DTR-PA after initially presenting with a less resistant phenotype and supported the evaluation of resistance progression. However, it may have influenced group allocation and the estimated associations. Classification based solely on the first eligible isolate could alter the composition of the study groups and would not identify all patients who subsequently developed DTR-PA during the study period. Future studies should assess the robustness of these findings using alternative isolate-selection strategies, including a first-isolate approach.

Third, detailed information on the specific antibacterial agents used and treatment duration within the previous 3 months was not consistently available for all patients. Therefore, prior antibiotic exposure was analyzed as a binary variable. Although this approach ensured a consistent exposure assessment across the study population, it did not capture cumulative treatment duration, the number or classes of antibiotics used, or the intensity of antimicrobial exposure. Consequently, dose–response relationships and class-specific effects could not be evaluated.

Fourth, the primary objective of this study was to identify risk factors associated with the development and progression of drug resistance; therefore, important clinical outcomes such as mortality, recurrence, and long-term prognosis were not evaluated. Consequently, the impact of DTR-PA on patient outcomes could not be assessed in the present study.

Fifth, only phenotypic resistance patterns were systematically analyzed; the molecular mechanisms underlying resistance development were not comprehensively investigated.

Future multicenter prospective studies with larger sample sizes are warranted to validate our findings. In addition, integrating whole-genome sequencing, resistance determinant profiling, and molecular epidemiological investigations may further elucidate the evolutionary pathways and transmission dynamics of DTR-PA.

## 5 Conclusion

Using three resistance phenotypes—CSPA, CRPA, and DTR-PA—this study systematically characterized the clinical features and risk factors associated with both resistance acquisition and resistance escalation in *P. aeruginosa*. Our findings suggest that the emergence and progression of resistance represent a stepwise evolutionary process driven by the interplay between healthcare-related exposures and host susceptibility factors. Healthcare-associated factors, including antimicrobial exposure and invasive procedures, appear to play a predominant role in the initial acquisition of resistance, whereas chronic comorbidities and indicators of severe illness are more strongly associated with progression to the DTR phenotype. Patients with recent antimicrobial exposure, invasive medical interventions, and underlying comorbid conditions constitute key high-risk populations for DTR-PA infection. By establishing a resistance continuum spanning CSPA, CRPA, and DTR-PA, this study provides a clinically relevant framework for understanding resistance evolution in *P. aeruginosa* and offers evidence to support early risk stratification, antimicrobial stewardship, and targeted infection prevention strategies. Continued resistance surveillance and timely intervention remain essential to limiting the emergence and dissemination of DTR-PA. 

## Data Availability

The original contributions presented in the study are included in the article/**Supplementary Material**. Further inquiries can be directed to the corresponding authors.
