# Supplementary material for: Clinical gradient and predictors for stepwise resistance escalation of carbapenem-susceptible, carbapenem-resistant, and difficult-to-treat resistant Pseudomonas aeruginosa infections: a 6-year retrospective cohort study
Source: Front Cell Infect Microbiol. 2026 Sep 14;16:1930952. doi: 10.3389/fcimb.2026.1930952 (PMC13616671; doi:10.3389/fcimb.2026.1930952)
Supplement: Supplementary file 1 [file Table1.docx]

**Supplementary Material**

**Supplementary Table S1**. Univariable logistic regression analysis of risk factors associated with DTR-PA versus non-DTR-PA.

| Variable | OR | Lower | Upper | ***p*** |
| --- | --- | --- | --- | --- |
| ICU stay within the previous 3 months | 4.666 | 2.449 | 8.890 | <0.001 |
| Soft tissue infection | 5.653 | 2.229 | 14.334 | <0.001 |
| CVC | 2.798 | 1.605 | 4.880 | <0.001 |
| Endotracheal intubation or tracheostomy | 2.702 | 1.561 | 4.675 | <0.001 |
| *Klebsiella pneumoniae* infection | 2.819 | 1.477 | 5.381 | 0.002 |
| Cerebrovascular disease | 2.742 | 1.457 | 5.162 | 0.002 |
| 3-month antibiotic exposure history | 2.432 | 1.388 | 4.262 | 0.002 |
| Urinary catheter | 2.473 | 1.381 | 4.427 | 0.002 |
| *Acinetobacter baumannii* infection | 2.551 | 1.346 | 4.833 | 0.004 |
| Lumbar puncture/Bone marrow aspiration | 4.407 | 1.583 | 12.266 | 0.005 |
| Diabetes | 2.290 | 1.231 | 4.259 | 0.009 |
| BMI | 0.923 | 0.863 | 0.988 | 0.022 |
| Corticosteroid | 0.431 | 0.202 | 0.917 | 0.029 |
| ALB | 0.953 | 0.913 | 0.996 | 0.032 |
| Chest drainage | 1.985 | 1.006 | 3.920 | 0.048 |
| Critically ill | 1.807 | 0.987 | 3.310 | 0.055 |
| Times of hospitalizations | 1.075 | 0.997 | 1.159 | 0.059 |
| Chronic kidney disease | 0.161 | 0.021 | 1.226 | 0.078 |
| SGLT2 inhibitor | 3.340 | 0.867 | 12.859 | 0.079 |
| Gender | 0.590 | 0.314 | 1.109 | 0.101 |
| Length of stay, >90 days | 3.660 | 0.770 | 17.400 | 0.103 |
| Urinary tract infection | 2.098 | 0.755 | 5.835 | 0.155 |
| Advanced antibiotics | 0.677 | 0.395 | 1.160 | 0.155 |
| Rheumatic autoimmune | 0.344 | 0.078 | 1.508 | 0.157 |
| TBIL | 0.984 | 0.960 | 1.010 | 0.220 |
| ICU admission during the current hospitalization | 1.376 | 0.802 | 2.361 | 0.246 |
| Tumour | 0.668 | 0.336 | 1.326 | 0.248 |
| Viral coinfection | 0.595 | 0.222 | 1.595 | 0.301 |
| COPD/Bronchiectasis | 1.504 | 0.689 | 3.282 | 0.305 |
| Length of stay, 14-90 days | 1.330 | 0.760 | 2.330 | 0.324 |
| Intra-abdominal infection | 1.827 | 0.543 | 6.152 | 0.329 |
| Ccr | 1.001 | 0.999 | 1.002 | 0.347 |
| Hospitalization within the previous 3 months | 1.304 | 0.734 | 2.316 | 0.364 |
| Cardiovascular disease | 1.244 | 0.707 | 2.188 | 0.447 |
| Trauma | 0.728 | 0.308 | 1.720 | 0.469 |
| ALT | 0.997 | 0.990 | 1.005 | 0.479 |
| TP | 0.991 | 0.965 | 1.017 | 0.486 |
| AST | 0.999 | 0.994 | 1.003 | 0.532 |
| Pulmonary infection | 1.207 | 0.655 | 2.227 | 0.545 |
| Bloodstream infection | 0.692 | 0.196 | 2.446 | 0.567 |
| Fungal coinfection | 1.191 | 0.421 | 3.365 | 0.741 |
| Age | 0.997 | 0.981 | 1.015 | 0.770 |
| Immunosuppressant | 1.000 | 0.206 | 4.850 | 1.000 |
| Central nervous system infection | 1.000 | 0.109 | 9.170 | 1.000 |

DTR-PA, difficult-to-treat resistant *Pseudomonas aeruginosa*; ICU, intensive care unit; CVC, Central venous catheterization; BMI, body mass index; ALB, albumin; SGLT2, sodium-glucose cotransporter 2; TBIL, total bilirubin; COPD, chronic obstructive pulmonary disease; Ccr, creatinine clearance rate; ALT, alanine aminotransferase; TP, total protein; AST, aspartate aminotransferase;

**Supplementary Table S2.** Univariable logistic regression analysis of risk factors associated with DTR-PA versus CRPA.

| Variable | OR | Lower | Upper | ***p*** |
| --- | --- | --- | --- | --- |
| ICU stay within the previous 3 months | 4.481 | 2.109 | 9.518 | <0.001 |
| Diabetes | 4.326 | 1.952 | 9.587 | <0.001 |
| Skin and soft tissue | 8.577 | 2.286 | 32.190 | 0.002 |
| 3-month antibiotic exposure history | 2.507 | 1.358 | 4.627 | 0.003 |
| Lumbar puncture/Bone marrow aspiration | 4.407 | 1.267 | 15.330 | 0.020 |
| *Klebsiella pneumoniae* infection | 2.256 | 1.101 | 4.622 | 0.026 |
| Urinary tract infection | 4.295 | 1.030 | 17.905 | 0.045 |
| SGLT2 inhibitor | 8.444 | 0.912 | 78.171 | 0.060 |
| Cardiovascular disease | 1.779 | 0.936 | 3.381 | 0.078 |
| CVC | 1.692 | 0.929 | 3.084 | 0.085 |
| Cerebrovascular disease | 1.768 | 0.895 | 3.490 | 0.100 |
| TBIL | 0.974 | 0.944 | 1.005 | 0.103 |
| COPD/Bronchiectasis | 2.175 | 0.853 | 5.551 | 0.103 |
| Urinary catheter | 1.684 | 0.896 | 3.165 | 0.105 |
| Times of hospitalizations | 1.076 | 0.984 | 1.176 | 0.107 |
| Corticosteroid | 0.516 | 0.228 | 1.163 | 0.110 |
| Advanced antibiotics | 0.617 | 0.341 | 1.118 | 0.111 |
| Tumour | 0.572 | 0.274 | 1.193 | 0.135 |
| Chronic kidney disease | 0.210 | 0.026 | 1.719 | 0.145 |
| ALT | 0.994 | 0.985 | 1.003 | 0.173 |
| Trauma | 0.563 | 0.227 | 1.396 | 0.214 |
| Hospitalization within the previous 3 months | 1.479 | 0.792 | 2.762 | 0.219 |
| Rheumatic autoimmune | 0.382 | 0.080 | 1.810 | 0.224 |
| *Acinetobacter baumannii* infection | 1.499 | 0.760 | 2.955 | 0.242 |
| Viral coinfection | 0.555 | 0.194 | 1.586 | 0.270 |
| ICU admission during the current hospitalization | 0.719 | 0.398 | 1.300 | 0.274 |
| BMI | 0.961 | 0.893 | 1.034 | 0.282 |
| Endotracheal intubation or tracheostomy | 1.349 | 0.746 | 2.440 | 0.320 |
| Length of stay, >90 days | 2.563 | 0.399 | 16.441 | 0.321 |
| TP | 1.015 | 0.985 | 1.045 | 0.336 |
| Gender | 0.729 | 0.365 | 1.454 | 0.367 |
| Length of stay, 14-90 days | 0.751 | 0.401 | 1.404 | 0.370 |
| Critically ill | 0.742 | 0.369 | 1.491 | 0.400 |
| AST | 0.997 | 0.989 | 1.004 | 0.401 |
| Bloodstream infection | 0.581 | 0.153 | 2.205 | 0.423 |
| Chest drainage | 1.322 | 0.638 | 2.741 | 0.451 |
| Age | 0.994 | 0.976 | 1.013 | 0.527 |
| Pulmonary infection | 0.812 | 0.407 | 1.621 | 0.554 |
| Fungal coinfection | 0.751 | 0.254 | 2.216 | 0.602 |
| Immunosuppressant | 0.656 | 0.128 | 3.377 | 0.613 |
| Intra-abdominal infection | 1.354 | 0.366 | 5.013 | 0.648 |
| Central nervous system infection | 0.662 | 0.067 | 6.576 | 0.723 |
| Ccr | 1.000 | 0.999 | 1.002 | 0.741 |
| ALB | 0.995 | 0.947 | 1.047 | 0.859 |

DTR-PA, difficult-to-treat resistant *Pseudomonas aeruginosa*; CRPA, carbapenem-resistant *P. aeruginosa*; ICU, intensive care unit; SGLT2, sodium-glucose cotransporter 2; CVC, Central venous catheterization; TBIL, total bilirubin; COPD, chronic obstructive pulmonary disease; ALT, alanine aminotransferase; BMI, body mass index; TP, total protein; AST, aspartate aminotransferase; ALB, albumin; Ccr, creatinine

**Supplementary Table S3.** Univariable logistic regression analysis of risk factors associated with R-PA versus CSPA

| Variable | OR | Lower | Upper | ***p*** |
| --- | --- | --- | --- | --- |
| Tracheostomy | 5.757 | 3.316 | 9.996 | <0.001 |
| Critically ill | 4.536 | 2.809 | 7.325 | <0.001 |
| ICU admission during the current hospitalization | 3.628 | 2.232 | 5.899 | <0.001 |
| CVC | 3.919 | 2.230 | 6.889 | <0.001 |
| ALB | 0.914 | 0.879 | 0.950 | <0.001 |
| Length of stay, 14-90 days | 2.735 | 1.731 | 4.321 | <0.001 |
| *Acinetobacter baumannii* infection | 4.807 | 2.185 | 10.576 | <0.001 |
| Urinary catheter | 2.929 | 1.617 | 5.304 | <0.001 |
| Cerebrovascular disease | 3.680 | 1.780 | 7.606 | <0.001 |
| TP | 0.957 | 0.934 | 0.981 | 0.001 |
| Chest drainage | 2.985 | 1.430 | 6.230 | 0.004 |
| BMI | 0.929 | 0.882 | 0.978 | 0.006 |
| Pulmonary infection | 1.942 | 1.198 | 3.150 | 0.007 |
| *Klebsiella pneumoniae* infection | 2.241 | 1.143 | 4.392 | 0.019 |
| ICU stay within the previous 3 months | 2.100 | 1.067 | 4.131 | 0.032 |
| Gender | 0.602 | 0.375 | 0.964 | 0.035 |
| Corticosteroid | 0.584 | 0.351 | 0.971 | 0.038 |
| Fungal infection | 3.197 | 1.053 | 9.705 | 0.040 |
| Cardiovascular disease | 0.629 | 0.394 | 1.003 | 0.051 |
| TBIL | 1.017 | 0.999 | 1.034 | 0.058 |
| Chronic kidney disease | 0.449 | 0.193 | 1.046 | 0.063 |
| Diabetes | 0.656 | 0.376 | 1.145 | 0.138 |
| Ccr | 1.003 | 0.999 | 1.008 | 0.144 |
| Immunosuppressant | 2.736 | 0.569 | 13.162 | 0.208 |
| Length of stay, >90 days | 2.923 | 0.545 | 15.669 | 0.210 |
| Intra-abdominal infection | 2.286 | 0.614 | 8.507 | 0.217 |
| Lumbar puncture/Bone marrow aspiration | 2.063 | 0.648 | 6.567 | 0.219 |
| Trauma | 1.504 | 0.763 | 2.964 | 0.238 |
| 3-month antibiotic exposure history | 1.271 | 0.818 | 1.974 | 0.285 |
| Rheumatic disease | 0.646 | 0.280 | 1.488 | 0.303 |
| COPD/Bronchiectasis | 0.712 | 0.361 | 1.406 | 0.327 |
| Skin and soft tissue nfection | 1.597 | 0.596 | 4.281 | 0.351 |
| Urinary tract infection | 0.651 | 0.251 | 1.691 | 0.377 |
| Central nervous system infection | 2.701 | 0.296 | 24.627 | 0.377 |
| Times of hospitalizations | 1.033 | 0.957 | 1.115 | 0.403 |
| Age | 1.005 | 0.991 | 1.019 | 0.466 |
| Tumour | 1.167 | 0.691 | 1.971 | 0.562 |
| Hospitalization within the previous 3 months | 0.878 | 0.555 | 1.389 | 0.578 |
| AST | 0.999 | 0.997 | 1.002 | 0.581 |
| ALT | 0.999 | 0.997 | 1.002 | 0.609 |
| Bloodstream infection | 1.255 | 0.485 | 3.242 | 0.639 |
| SGLT2 inhibitor | 0.829 | 0.217 | 3.161 | 0.783 |
| Advanced antibiotics | 1.020 | 0.656 | 1.586 | 0.929 |
| Viral coinfection | 0.975 | 0.485 | 1.961 | 0.943 |

CSPA, carbapenem-susceptible *Pseudomonas aeruginosa*; R-PA, resistant *P. aeruginosa* (including CRPA and DTR-PA); ICU, intensive care unit; CVC, Central venous catheterization; ALB, albumin; TP, total protein; BMI, body mass index; TBIL, total bilirubin; Ccr, creatinine; COPD, chronic obstructive pulmonary disease; AST, aspartate aminotransferase; ALT, alanine aminotransferase; SGLT2, sodium-glucose cotransporter
